# Age-related changes in antigen-specific natural antibodies are influenced by sex

**DOI:** 10.3389/fimmu.2022.1047297

**Published:** 2023-01-12

**Authors:** Sarah E. Webster, Naomi L. Tsuji, Michael J. Clemente, Nichol E. Holodick

**Affiliations:** ^1^ Center for Immunobiology, Department of Investigative Medicine, Western Michigan University Homer Stryker M.D. School of Medicine, Kalamazoo, MI, United States; ^2^ Flow Cytometry and Imaging Core, Center for Immunobiology, Department of Investigative Medicine, Western Michigan University Homer Stryker M.D. School of Medicine, Kalamazoo, MI, United States

**Keywords:** B-1 cell, natural antibody, phosphatidylcholine, aging, biological sex

## Abstract

**Introduction:**

Natural antibody (NAb) derived from CD5+ B-1 cells maintains tissue homeostasis, controls inflammation, aids in establishing long-term protective responses against pathogens, and provides immediate protection from infection. CD5+ B-1 cell NAbs recognize evolutionarily fixed epitopes, such as phosphatidylcholine (PtC), found on bacteria and senescent red blood cells. Anti-PtC antibodies are essential in protection against bacterial sepsis. CD5+ B-1 cell-derived NAbs have a unique germline-like structure that lacks N-additions, a feature critical for providing protection against infection. Previously, we demonstrated the repertoire and germline status of PtC+CD5+ B-1 cell IgM obtained from male mice changes with age depending on the anatomical location of the B-1 cells. More recently, we demonstrated serum antibody from aged female mice maintains protection against pneumococcal infection, whereas serum antibody from male mice does not provide protection.

**Results:**

Here, we show that aged female mice have significantly more splenic PtC+CD5+ B-1 cells and more PtC specific serum IgM than aged male mice. Furthermore, we find both age and biological sex related repertoire differences when comparing B cell receptor (BCR) sequencing results of PtC+CD5+ B-1 cells. While BCR germline status of PtC+CD5+ B-1 cells from aged male and female mice is similar in the peritoneal cavity, it differs significantly in the spleen, where aged females retain germline configuration and aged males do not. Nucleic acid sensing toll-like receptors are critical in the maintenance of PtC+ B-1 cells; therefore, to begin to understand the mechanism of differences observed between the male and female PtC+CD5+ B-1 cell repertoire, we analyzed levels of cell-free nucleic acids and found increases in aged females.

**Conclusion:**

Our results suggest the antigenic milieu differs between aged males and females, leading to differential selection of antigen-specific B-1 cells over time. Further elucidation of how biological sex differences influence the maintenance of B-1 cells within the aging environment will be essential to understand sex and age-related disparities in the susceptibility to bacterial infection and will aid in the development of more effective vaccination and/or therapeutic strategies specific for males and females.

## 1 Introduction

Natural antibodies are polyreactive, low affinity immunoglobulins of varying isotypes found in both humans and mice (1–3). Specifically, IgM specific natural antibodies provide several essential functions including protection from infection (1), regulation of B cell development (4–6), selection of the B cell repertoire (5–7), clearance of apoptotic debris (1), protection against atherosclerosis (8, 9), and allergic suppression (10). In many diseases associated with aging including atherosclerosis (11–13), cancer (14), stroke (15), Alzheimer’s disease (16), influenza (17), and pneumococcal infection (18) natural antibodies have been demonstrated to be protective. In mice, 80-90% of natural IgM is produced by B-1 cells (2–4).

Phenotypic and functional analyses have identified different subsets of B cells, which are broadly categorized as B-1 and conventional B2 cells. While B2 cells are generally understood to play a major role in adaptive immunity, B-1 cells seem to exist in the margin between adaptive and innate immunity. Most circulating natural IgM is derived from B-1 cells (2–4), which arise early in life and persist into adulthood *via* self-renewal (19). B-1 cells were originally identified by expression of CD5 and were further characterized by surface expression of IgM^high^, IgD^low^, CD19^high^, B220^low^, CD23-, and CD43+ (20), which contrasts with the surface phenotype of follicular B-2 cells: CD5-, IgM^low^, IgD^high^, CD19+, B220+, CD23+, and CD43-. Later, an additional population of B-1 cells was identified sharing the characteristics of CD5+ B-1 but lacking CD5 expression (21). Recent literature indicates that CD5+ cells lose CD5- expression upon multiple rounds of cell division (22). Given that CD5- B-1 cells appear to derive from CD5+ B-1 cells (22), examination of antigen specific CD5+ B-1 cells is representative of the available natural antibody repertoire. Herein, we examine CD5+ antigen specific B-1 cells to understand how age and biological sex influences antigen specific natural antibody.

The B-1 cell compartment changes with increasing age (23, 24), and, as recently demonstrated, is also influenced by biological sex (25). Examination of the B-1 cell repertoire has shown that unlike conventional B2 cells, the structure of natural IgM is germline-like due to minimal insertion of non-template-encoded N nucleotides (N-region additions) along with little evidence of somatic hypermutation (26, 27). This germline-like nature of B-1 cell derived natural IgM is required for protection against *Streptococcus pneumoniae* infection (28), and is lost in aged males (23, 25). In contrast to aged males, B-1 cell derived natural IgM obtained from aged females retains germline-like status as well as the ability to protect against *S. pneumoniae* infection (25). In males, age-related changes seen in natural antibodies are dependent upon antibody specificity and anatomical location (24), however, it is unknown if such changes occur similarly in aged females.

Approximately 5-15% of peritoneal CD5+ B-1 cells are specific for phosphatidylcholine (PtC), an antigen found on senescent red blood cells as well as bacterial cell membranes (29, 30). Anti-PtC antibodies have been shown to be essential in protection from bacterial sepsis (31). Importantly, biological sex differences are observed in the mortality of patients with sepsis (32–35). Considering our previous studies and the extensive literature demonstrating the major role selection plays in shaping the CD5+ B-1 cell pool, we questioned how PtC specific (PtC+) CD5+ B-1 cells would be affected during aging in female mice. To examine this, we performed single-cell BCR sequencing on PtC+CD5+ B-1 cells from young and aged female BALB/c-ByJ mice. Here, we find differences in the selection of PtC+CD5+ B-1 cells in females versus males over time, demonstrating that aged males utilize different BCR specificities than aged females. The sex-related differences in the natural antibody repertoire observed in our studies have implications for susceptibility to infection and disparities between men and women in the incidence and/or death rate of many diseases of the aged (36).

## 2 Materials and methods

### 2.1 Mice

Male and female BALB/cByJ mice were obtained from The Jackson Laboratory at 6–8 weeks of age and aged in our vivarium until the age indicated. The mice were housed at 5 mice per cage with a 12-hour light/12-hour dark cycle and ad libitum access to water and food. Mice were cared for and handled in accordance with the Guide for the Care and Use of Laboratory Animals, National Institutes of Health, and institutional guidelines. All animal studies were approved by the Western Michigan University Homer Stryker M.D. School of Medicine IACUC committee.

### 2.2 Cell purification and flow cytometry

Peritoneal lavage and spleen removals were performed on all euthanized mice. Spleens were homogenized using the rough ends of glass slides or the Miltenyi gentleMACS dissociator and then passed through a 70-μm cell strainer. All samples were treated with RBC lysis buffer for 2 minutes (Lonza), subsequently diluted with HBSS with 2.5% FBS, and then centrifuged at 1200rpm for 10 minutes. The cells were resuspended in HBSS with 2.5% FBS, stained with immunofluorescent antibodies, and then analyzed on a LSR Fortessa flow cytometer or Influx cell sorter (BD Biosciences) with gating on live cells by forward side scatter and/or Aqua Live/Dead stain (Invitrogen). Images were constructed with FlowJo™ v10.6.2 Software (BD Life Sciences). The following antibodies were obtained from BD Pharmingen: CD19 (clone ID3), CD43 (clone S7), B220/CD45 (clone RA3-6B2), CD23 (clone B3B4), CD5 (clone 53-7.3). FITC labeled PtC liposomes (purchased from Dr. Aaron Kantor), diluted at 1:30,000, were used to detect PtC+ B cells, as previously described (5, 24). The composition of the PtC liposomes used is DSPC : DSPG : Chol (Molar ratio: 45:5:50).

### 2.3 Single-cell sequencing and analysis

Peritoneal washout cells and splenocytes were obtained from BALB/c-ByJ mice at the indicated age and stained with fluorescence-labeled antibodies. PtC+CD5+ B-1 cell populations were single-cell sorted using an Influx cell sorter (BD Biosciences) into a 96-well plate containing lysis buffer (RNaseOut, 5X Buffer, DTT, IgePAL, carrier RNA, Invitrogen). Post-sort re-analysis of CD5+ B-1 cell populations showed them to be ≥;98% pure. To obtain cDNA, a 20μl reverse transcription reaction was run per well using the SuperScript III enzyme and random hexamers (Invitrogen). Qiagen’s HotStart Taq Plus master mix kit was used to perform the first round of PCR (25μl reaction) using 2.5μl of cDNA diluted 1:2 and the following primers: MsV_H_E and MsCμE each at 0.6μM, as previously described (24, 27). Each 25μl reaction was run as follows: 95°C for 5 minutes; 35 cycles at 94°C for 30 seconds, 50°CCfor 30 seconds, 72°C for 30 seconds; and then a final extention at 72°C for 10 minutes. The product from this first reaction was then diluted at 1:100 in dH2O and 2μl was used in the second semi-nested 25μl reaction using the following primers: MsV_H_E and MsCμN each at 0.6μM, as previously described (24, 27). The second reaction was run as follows: 95°C for 5 minutes; 40 cycles at 94°C for 30 seconds, 53°C for 30 seconds, 72°C for 30 seconds; and then a final extension at 72°C for 10 minutes. The products were run on the Qiagen Qiaxcel. PCR products were sequenced (Genewiz) using the MsV_H_E primer. Sequences were analyzed using an online sequence analysis tool, IMGT/HighV-Quest (37).

### 2.4 DMPC ELISA analysis

Serum was collected from individual female BALB/c-ByJ naïve mice at the time of euthanasia at the ages indicated. The serum was analyzed for antibody against DMPC by ELISA. ELISA strips were obtained from Avanti Polar Lipids pre-coated with 1,2-dimyristoyl-sn-glycero-3-phosphocholine (DMPC). Wells were blocked with 200μl of 3% fatty acid free bovine serum albumin in PBS for one hour at room temperature with gentle shaking and then washed three times with 1X PBS. Diluted serum was added at 50μl per well and incubated for one hour at room temperature with gentle shaking. The wells were then washed three times with 1X PBS. Bound antibody was measured using HRP-conjugated goat anti-mouse IgM (Bethyl Labs) at 1:20,000. NC-17, kindly provided by Dr. Gregg Silverman, was used as a standard and included on each plate.

### 2.5 Cell-free nucleic acid extraction from serum

Serum was collected from individual young (3- to 4-mo) and old (18- to 24-mo) male and female mice and processed with the Quick-cfDNA/cfRNA Serum & Plasma kit (Zymo Research) according to the manufacturer’s directions. Briefly, serums were centrifuged at 16,000 g for 10 minutes to remove any cell debris and precipitates before undergoing Proteinase K digestions. Nucleic acids were extracted from 200 μL of serum. Samples were run through the Quick-cfDNA/cfRNA columns and cfDNA/cfRNA was collected through the co-purification method. Extracted cfDNA/RNA was stored at -80°C prior to further analysis.

### 2.6 Cell-free nucleic acid fragment size and concentration

Extracted cell-free nucleic acids from each individual serum sample were quantified using the dsDNA high sensitivity assay, ssDNA assay, and RNA high sensitivity assay for the Qubit 4 (ThermoFisher) as well as on the 2100 Bioananalzyer (Agilent Technologies) with HS DNA chips for assessment of sample purity, concentration, and fragment size distribution according to the manufacturer’s instructions. The average fragment size was determined with the Agilent 2100 Bioanalzyer Expert software and calculated across the first three major peaks 50 – 650 base pairs (bp) corresponding to the length of nucleosomal footprints derived from apoptotic cells while high molecular weight cfDNA was calculated between 2000 – 3000 bp corresponding to cfDNA derived from necrotic cells (38–40). The final serum cfDNA/RNA concentration were calculated by adjusting for the initial serum and final elution volumes and quantified with the Qubit 4.

### 2.7 Statistics

Statistical analyses were performed using Prism (Version 9.0). All statistical analyses used are indicated in each figure legend. The outlier test was performed on all data sets using Prism’s ROUT method of identifying outliers. Outliers were removed when detected by Prism’s ROUT method using the coefficient Q set at 1%. Error reported as standard error of the mean.

## 3 Results

### 3.1 Aged female mice display an increase in splenic PtC specific CD5+ B-1 cells and serum PtC specific IgM as compared to aged male mice

**Figure 1:**
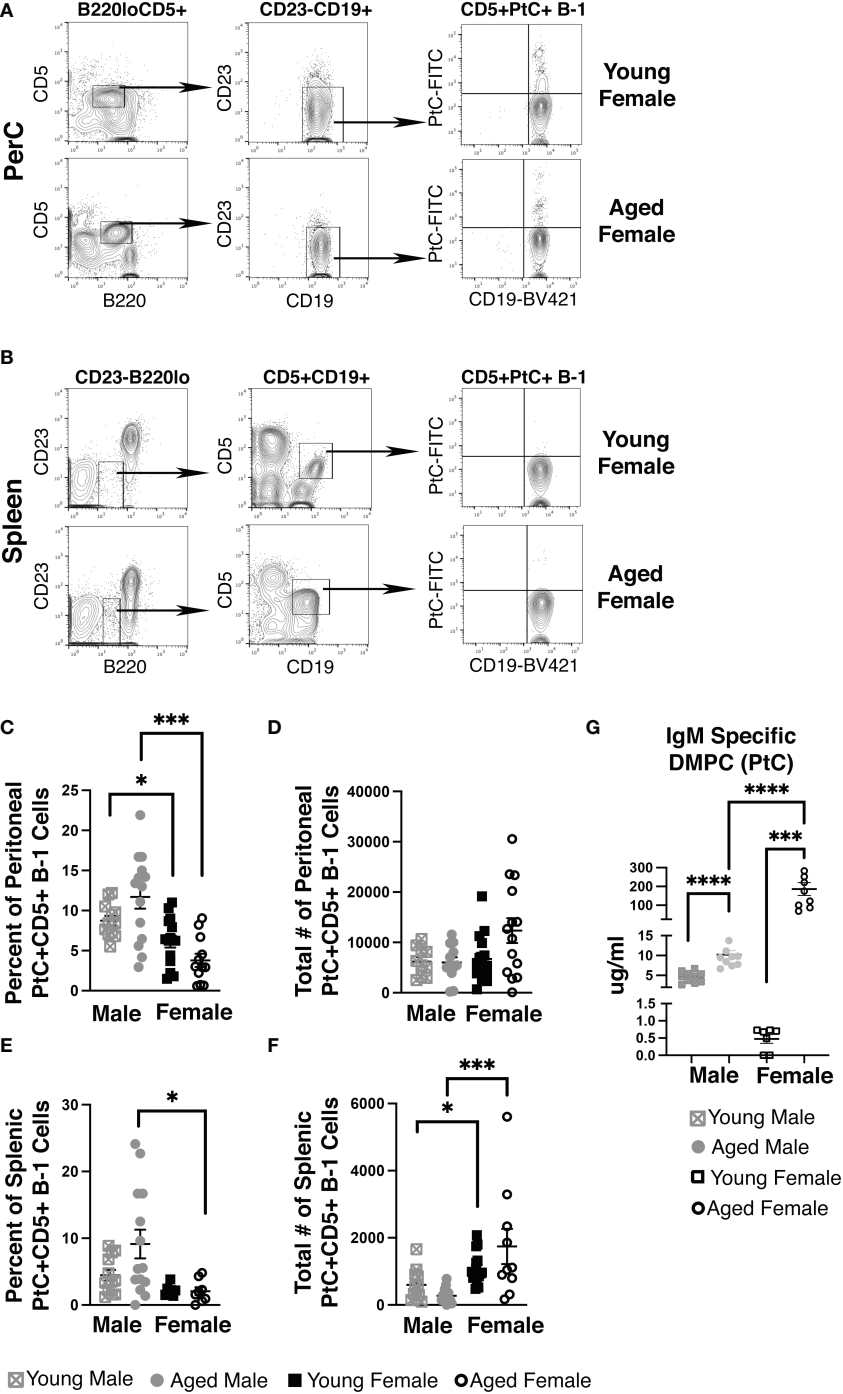
Female mice have an increase in PtC-specific splenic CD5+ B-1 cells and serum anti-DMPC antibody. Representative gating strategy for **(A)** peritoneal cavity and **(B)** splenic PtC+CD5+ B-1 cells. The frequency **(C**, **E)** and absolute number **(D, F)** of PtC+CD5+ B-1 cells were assessed in young (3-month-old) and aged (18-22-month-old) male and female BALB/c-ByJ mice (young male n=12, aged male n=14, young female n=15, aged female n=14). **(C)** The percent of live peritoneal lymphocytes staining positive for PtC+CD5+ B-1 cells (B220^lo^CD5^+^CD19^hi^CD23^-^PtC^+^), **(D)** total number of peritoneal PtC+CD5+ B-1 cells, **(E)** the the percent of live splenocytes staining positive for PtC+CD5+ B-1 cells (B220^lo^CD5^+^CD19^hi^CD23^-^PtC^+^), and **(F)** total number of splenic PtC+CD5+ B-1 cells. **(G)** Serum levels of DMPC-specific IgM from young (3-month old) and aged (22-26-month old) mice (young male n=10, aged male n=10, young female n=7, aged female n=9). Grey squares represent young male mice, grey circles represent aged male mice, open black squares represent young female mice, and open black circles represent aged female mice. All results are based on 3 independent experiments. Values are displayed as the mean ( ± SEM) of individual mouse serum samples. Statistics used: Mann-Whitney test. Asterisks for p values: *p<0.05, ***p<0.001.

Using fluorescently labeled PtC-liposomes (41), we assessed the female peritoneal (PerC) and splenic CD5+ B-1 cell pools for PtC specificity (representative plots in **Figures 1A, B**). While the frequency of PerC CD5+ B-1 cells that bound PtC-liposomes did not change significantly in age-grouped female mice (mean of 3.8%±0.8 in aged vs. 6.2%±0.8 in young) the frequency of PerC PtC+CD5+ B-1 cells is significantly higher in both young and aged males as compared to young and aged females, respectively (**Figure 1C**). The absolute number of PerC PtC+CD5+ B-1 cells shows no significant difference (**Figure 1D**). In the spleen, the frequency of PtC+CD5+ B-1 cells is significantly higher in aged males as compared to aged females (**Figure 1E**); however, the absolute number of splenic PtC+CD5+ B-1 cells is significantly higher in aged females as compared to aged males (**Figure 1F**). The differences in percent but not total number of PtC specific B-1 cells is due to differences in total CD5+ B-1 cell numbers in the peritoneal cavity and spleen (**Supplemental Figure 1**).

To test serum levels of PtC-specific antibody in aged females, we analyzed serum from young and aged mice by ELISA for IgM antibody that recognizes PtC (DMPC, 1,2-dimyristoyl-sn-glycero-3-phosphocholine is the phosphatidylcholine used to coat the ELISA plate). Interestingly, we observed significantly more DMPC specific IgM in serum from aged female mice as compared to young female mice (p=0.0079) (**Figure 1G**). Furthermore, young female mice have significantly less serum DMPC specific IgM levels than young male mice (0.80 μg/ml ±0.2 vs. 4.58 μg/ml ±0.43, *p=0.0007*). Aged female mice have significantly more serum DMPC specific IgM levels than aged male mice (189 μg/ml ±40.1 vs. 10.2 μg/ml ±1.06, *p=0.0007*) (**Figure 1G**). These results demonstrate in both male and female mice, serum PtC-specific antibody increases with age; however, aged female mice have significantly more serum PtC-specificIgM than aged males.

### 3.2 Repertoire of PtC specific peritoneal and splenic CD5+ B-1 cell changes in aged females

**Figure 2:**
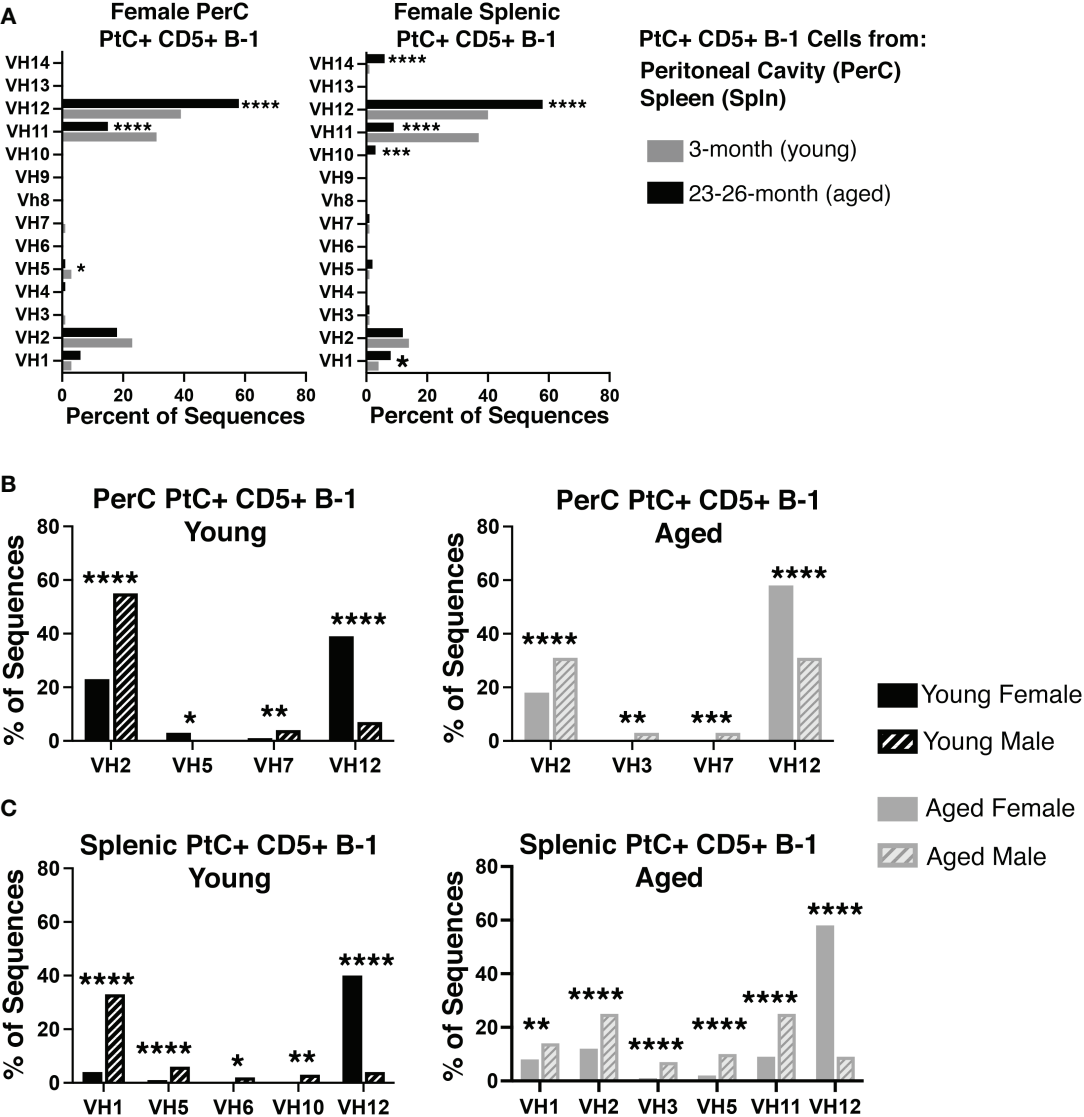
Repertoire analysis of natural IgM from peritoneal and splenic PtC+ CD5+ B-1 cells in adult female young and aged mice. PtC+CD5+ B-1 cells were single-cell sorted from the peritoneal cavity or spleen of 3- and 23-26-month-old female BALB/c-ByJ mice. The V_H_ region was amplified and sequenced as detailed in Materials and Methods. **(A)** The percent of V_H_ gene segment usage (young in grey bars, aged in black bars). **(B, C)** For direct comparison of females and males,previously published (24) repertoire analyses of young and aged male PtC+CD5+ B-1 cells is included. Results are based on 4 independent experiments with sequences combined from each independent experiment (n=11 for 3-month-old mice, n=15 for 23-26-month-old female mice). Statistics used: 2x2 chi-square test. Asterisks for p values: *p<0.05, **p<0.01, ***p<0.001, ****p<0.0001.

Considering aged females display considerably higher levels of PtC-specific serum antibody, we hypothesized that the aged female repertoire of PtC+CD5+ B-1 cells might be distinct from that of young females and aged males. Examination of the variable (V_H_), diversity (D_H_), and joining (J_H_), gene segments of the immunoglobulin heavy chain as well as germline status of PtC+CD5+ B-1 cells obtained from either the peritoneal cavity (PerC) or spleen of young and aged female mice revealed differences in 1) the germline status and 2) the V_H_, D_H_, and J_H_ gene usage between young and aged females. Aged PerC PtC+CD5+ B-1 cells utilized V_H_12 (58% vs. 39%) more frequently than young. In contrast, the young PerC PtC+CD5+ B-1 cells used V_H_5 (3% vs. 1%) and V_H_11 (31% vs. 15%) more frequently than aged. Aged splenic PtC+CD5+ B-1 cells used V_H_1 (8% vs. 4%), V_H_10 (3% vs. 0%), V_H_12 (58% vs. 40%), and V_H_14 (6% vs. 1%) more frequently than young whereas, the young splenic PtC+CD5+ B-1 cells used V_H_11 more frequently than aged (37% vs. 9%) (**Figure 2A**).

When female PtC+CD5+ B-1 cell repertoire data is compared to our previously published male PtC+CD5+ B-1 cell data, numerous significant differences were observed between male and female V_H_ utilization (**Figure 2B, C**). Young female PerC PtC+ CD5+ B-1 cells utilized V_H_12 and V_H_5 more frequently than young males whereas, young males utilized V_H_2 and V_H_7 more frequently than young females (**Figure 2B**). Aged female PerC PtC+CD5+ B-1 cells utilized V_H_12 more frequently than aged males, whereas aged males used V_H_2, V_H_3, and V_H_7 more frequently (**Figure 2B**). In the spleen, young and aged female PtC+CD5+ B-1 cells utilized V_H_12 more frequently than young and aged males (**Figure 2C**). However, young male splenic PtC+CD5+ B-1 cells used V_H_1, V_H_5, V_H_6, and V_H_10 more frequently than young females. In the aged spleen, male PtC+CD5+ B-1 cells utilized V_H_1, V_H_2, V_H_3, V_H_5, and V_H_11 more frequently than aged females. Together these results demonstrate significant differences in PtC+CD5+ B-1 cell V_H_ usage associated with biological sex and age.

**Figure 3:**
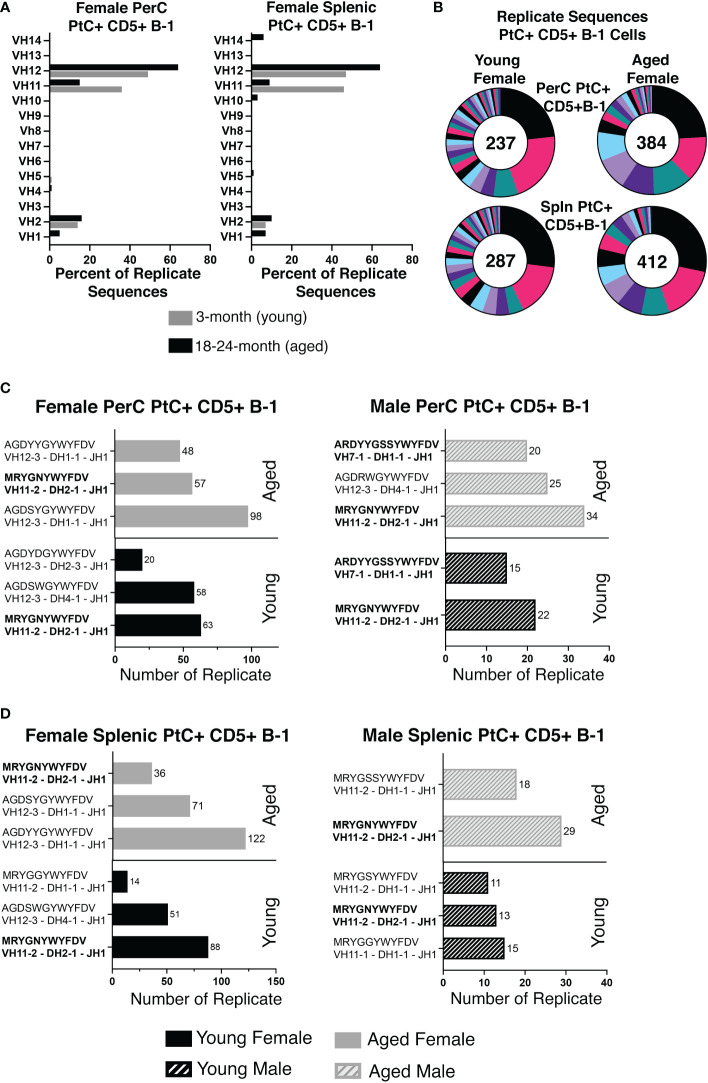
Significant differences in female versus male peritoneal and splenic PtC+CD5+ B-1 cell CDR-H3 use. PtC+CD5+ B-1 cells were single-cell sorted from the peritoneal cavity or spleen of young and aged female BALB/c-ByJ mice (as presented in **Figure 2**). **(A)** The percent of VH gene segment usage within the replicate sequences. **(B)** Distribution of replicate CDR-H3 sequences in the young and aged (number in the middle represents the number of replicates within the population). Each color represents a unique CDR-H3 amino acid sequence. **(C, D)** For direct comparison of females and males, previously published (24) CDR-H3 analysis of young and aged malePtC+CD5+ B-1 cells is included. **(C)** Comparison of the most frequently utilized CDR-H3 sequences of peritoneal PtC+CD5+ B-1 cells from young and aged male and female mice. **(D)** Comparison of the most frequently utilized CDR-H3 sequences of splenic PtC+CD5+ B-1 cells from young and aged male and female mice. Results are based on 4 independent experiments with sequences combined from each independent experiment (n=11 for 3-month-old mice, n=15 for 23-26-month-old female mice). Statistics used: 2x2 chi-square tests.

While heavy chain sequencing of complementary determining region 3 (CDR-H3) alone is not sufficient to establish clonal expansion, a higher proportion of replicate sequences (sequences with the exact same CDR-H3 sequence) suggests the presence of clonally divided cells. Sequences from young PerC PtC+CD5+ B-1 cells were replicated at 67% (237/354), while PerC PtC+CD5+ B-1 cells from aged mice were replicated at 85% (412/484) (**Figure 3A**). Similar results were found in the spleen, 69% (287/417) replicates from young vs. 85% (412/484) from aged (**Figure 3A**). Of the replicates observed in aged PerC and splenic PtC+CD5+ B-1 cells, V_H_12 was the most abundantly used V_H_ gene segment (**Figure 3A**). Furthermore, we observed a decrease in diversity, defined as the proportion of unique CDR-H3 sequences, within replicate sequences from aged female PerC and splenic PtC+CD5+ B-1 cells (**Figure 3B**). Quantification of the most utilized CDR-H3 sequences found the PtC/PC (phosphorylcholine) cross-reactive CDR-H3 (MRYGNYWYFDV, VH11) as the most frequently utilized replicate in both young female and male PerC and splenic PtC+CD5+ B-1 cells (**Figures 3C, D**). However, aged female PerC and splenic PtC+CD5+ B-1 cells utilize V_H_12 more frequently in replicates whereas, aged males utilize V_H_11 (**Figures 3C, D**). Splenic PtC+CD5+ B-1 cells from aged female mice are more diverse in their CDR-H3 replicate sequences (**Figure 3**) than splenic PtC+CD5+ B-1 cells from aged males (24); however, PerC PtC+CD5+ B-1 cells from both aged females and males are similar in replicate diversity.

**Figure 4:**
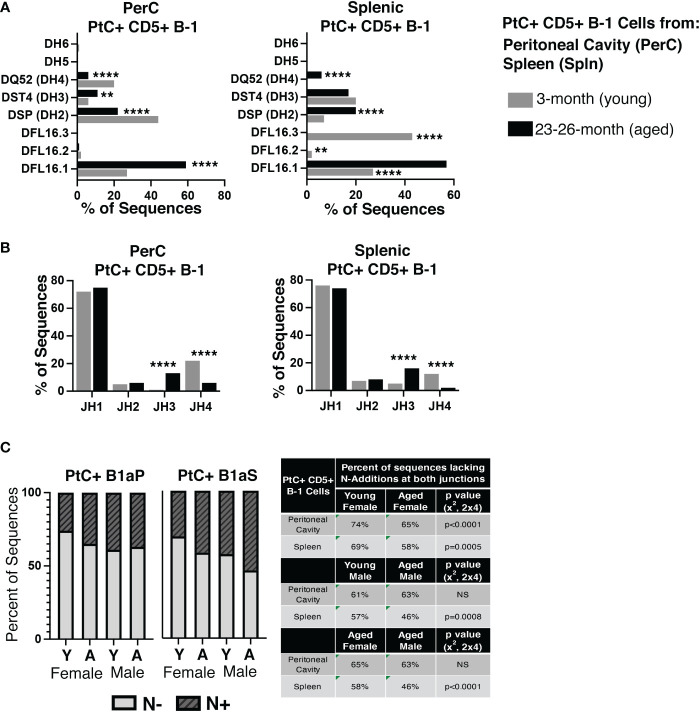
Analysis of D_H_ use, J_H_ use, and germline status of young and aged PtC+CD5+ B-1 cells. PtC+CD5+ B-1 cells were single-cell sorted from the peritoneal cavity or spleen of young and aged female BALB/c-ByJ mice (as presented in **Figure 2**). **(A)** The percent of D_H_ gene segment usage. **(B)** The percent of J_H_ gene segment usage. **(C)** The percent of sequences containing zero N-additions (light grey bars) or 1 or more N-additions (dark grey hashed bars) at both junctions is shown with replicate sequences included in the analysis. For direct comparison of females and males, previously published (24) N- addition analysis of young and aged male PtC+ CD5+ B-1 cells is included. Results are based on 4 independent experiments with sequences combined fromeach independent experiment (n=11 for 3-month-old mice, n=15 for 23-26-month-old female mice). Statistics used: 2x2 and 2x4 chi-square test. Asterisks for p values: **p<0.01, ****p<0.0001.

Examination of D_H_ and J_H_ genes show significant differences in utilization in the aged versus young female PerC and splenic PtC+ populations (**Figures 4A, B**), the details of which are summarized in **Supplemental Figure 2**. Of note, aged female PerC and splenic PtC+CD5+ B-1 cells utilized DFL16.1 (D_H_1-1) more frequently than young PtC+CD5+ B-1 (**Figure 4A**). The germline sequence of DFL16.1 has been shown to be essential in protection against pneumococcal infection (42).

The number of BCR sequences lacking N-additions at both female junctions (germline-like structure) changes with age in both female PerC PtC+CD5+ B-1 cells (65% in aged vs. 74% in young, *p<0.0001*, x2, 2x4) and splenic PtC+CD5+ B-1 cells (58% in aged vs. 0 69% in young, *p=0.0005*, x2, 2x4) (**Figure 4C**). Interestingly, there is no significant difference in the number of PerC PtC+CD5+ B-1 cell sequences lacking N-additions between aged female (65%) and aged male mice (63%); however, there is a significant difference in the number of splenic PtC+CD5+ B-1 cell sequences lacking N-additions between aged female mice (58%) as compared to aged male mice (46%) (*p<0.0001*, x2, 2x4) (**Figure 4C**). Together, these results demonstrate significant changes in the repertoire of both peritoneal and splenic PtC specific natural IgM obtained from aged female mice as compared to young female mice. Furthermore, these differences observed in female mice differ from previously published results examining PtC-specific natural IgM from aged male mice as compared to young male mice (24).

### 3.3 Hydrophobicity of the CDR-H3 loop changes with age, sex, and VH usage

**Figure 5:**
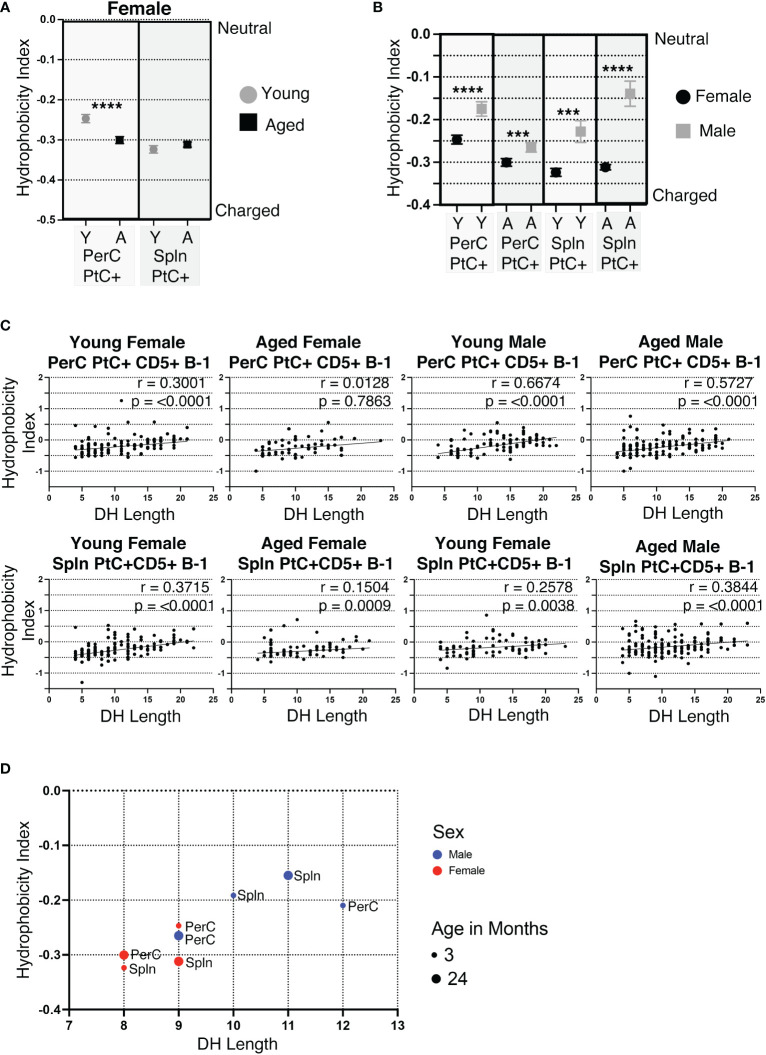
CDR-H3 hydrophobicity of PtC+CD5+ B-1 cell IgM changes with age and sex. PtC+CD5+ B-1 cells were single-cell sorted from the peritoneal cavity or spleen of young and aged female BALB/c-ByJ mice (as presented in **Figure 2**). “Y” indicates young mice (3-month-old) and “A” indicates aged (23-26-month-old) mice. **(A)** The average charge of the CDR-H3 loop region of IgM from the peritoneal or splenic PtC+CD5+ B-1 cells. **(B-D)** For direct comparison of females and males, previously published (24) hydrophobicity analysis of young and aged male PtC+CD5+ B-1 cells is included. **(B)** Female and male comparison of the average charge of the CDR-H3 loop region of IgM from peritoneal or splenic PtC+CD5+ B-1 cells. **(C, D)** Correlation of average charge of the CDR-H3 loop region with DH length. Results are based on 4 independent experiments with sequences combined from each independent experiment (n=11 for 3-month-old mice, n=15 for 23-26-month-old female mice). Statistics used: Mann-Whitney test for **(A, B)** and Spearman’s rank correlation coefficient **(C)**. Asterisks for p values: ***p<0.001, ****p<0.0001.

Of the 6 complementary determining regions (CDRs) in antibodies, CDR-H3 is the central point for antigen contact and the most variable in nucleotide/amino acid sequence. Hydrophobicity is a measurable characteristic of the CDR-H3. Hydrophobic CDR-H3 loops have been shown to be important for certain broadly neutralizing antibodies against HIV (43), whereas antibodies with highly charged CDR-H3 loops are often autoreactive (44, 45). We calculated the average hydrophobicity of each CDR-H3 loop using the Kyte-Doolittle scale. Our results demonstrate the CDR-H3 loop of peritoneal (PerC) PtC+CD5+ B-1 cell IgM increases in charge (decreases in hydrophobicity) with age (-0.247 ±0.010 in young vs. -0.300 ±0.009 in aged, *p<0.0001*) (**Figure 5A**). Furthermore, females have more highly charged CDR-H3 loops than males in both the PerC and splenic compartments (**Figure 5B**).

The CDR-H3 loop of B-1 cells is characteristically hydrophobic (46). D_H_ use has been shown to be primarily responsible, with D_H_ length positively correlating with increased hydrophobicity of CDR-H3 (46). In concurrence, the hydrophobicity of PerC and splenic PtC+CD5+ B-1 cell CDR-H3 loops positively correlate with the length of the respective D_H_ gene (**Figure 5C, D**). Young and aged male mice tend to utilize longer D_H_ gene segments than females, which correlates with the more hydrophobic CDR-H3 loops seen in males as compared to the more charged CDR-H3 loops in females (**Figures 5C, D**).

**Figure 6:**
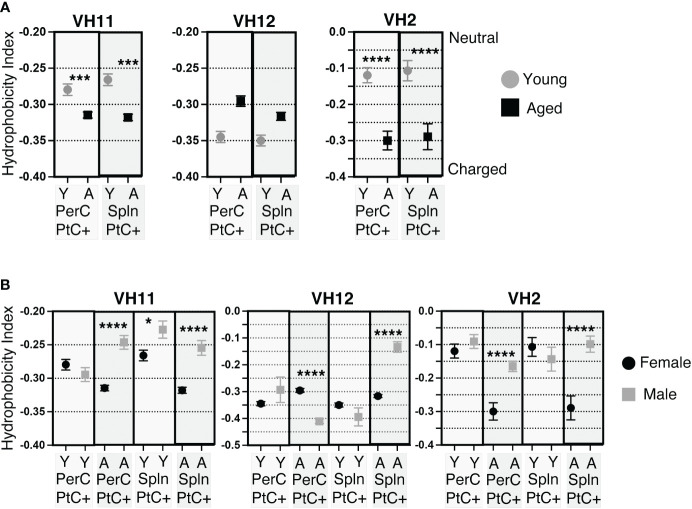
CDR-H3 hydrophobicity of PtC+CD5+ B-1 cell IgM utilizing V_H_11, V_H_12, or V_H_2. PtC+CD5+ B-1 cells were single-cell sorted from the peritoneal cavity or spleen of young and aged female BALB/c-ByJ mice (as presented in **Figure 2**). “Y” indicates young mice (3-month-old) and “A” indicates aged (23-26-month-old) mice. **(A)** The average charge of the CDR-H3 loop region of IgM utilizing either V_H_11, V_H_12, or V_H_2 from peritoneal or splenic PtC+CD5+ B-1 cells. **(B)** For direct comparisonof females and males, previously published (24) hydrophobicity analysis of young and aged male PtC+CD5+ B-1 cells is included. Female and male comparison of the average charge of the CDR-H3 loop region of IgM utilizing either V_H_11, V_H_12, or V_H_2 from peritoneal or splenic PtC+CD5+ B-1 cells. Results are based on 4 independent experiments with sequences combined from each independent experiment (n=11 for 3-month-old mice, n=15 for 23-26-month-old female mice). Statistics used: Mann-Whitney test. Asterisks for p values: *p<0.05, ***p<0.001, ****p<0.0001.

Previous studies have shown PtC specific B-1 cells most frequently utilize V_H_2, V_H_11, and V_H_12 (47, 48). Examination of CDR-H3 hydrophobicity of PtC specific B-1 cells utilizing V_H_2, V_H_11, or V_H_12 reveals sex and age-related differences. In female PerC and splenic PtC+CD5+ B-1 cells utilizing V_H_11 and V_H_2, there is a significant increase in charge with age (**Figure 6A**). Young female cells utilizing V_H_2 were more hydrophobic than those utilizing V_H_11 and V_H_12. The hydrophobicity of cells using V_H_12 did not change with age (**Figure 6A**). There were no significant differences in the hydrophobicity of PtC specific B-1 cells utilizing V_H_2, V_H_11, or V_H_12 between young female and male mice (**Figure 6B**). However, aged females displayed significantly higher charged CDR-H3 loops than aged males for V_H_2, V_H_11, and V_H_12 with one exception; PerC PtC+ B-1 cells utilizing VH12 from aged males were significantly increased in charge as compared to aged females (**Figure 6B**).

Together, these results demonstrate differences in hydrophobicity of the CDR-H3 loop in female PtC specific CD5+ B-1 cell populations with age as well as differences between aged male and female mice. Overall, female mice display more highly charged CDR-H3 loops than males.

### 3.4 Amino acid composition of the CDR-H3 loop changes with age, sex, and VH usage

**Figure 7:**
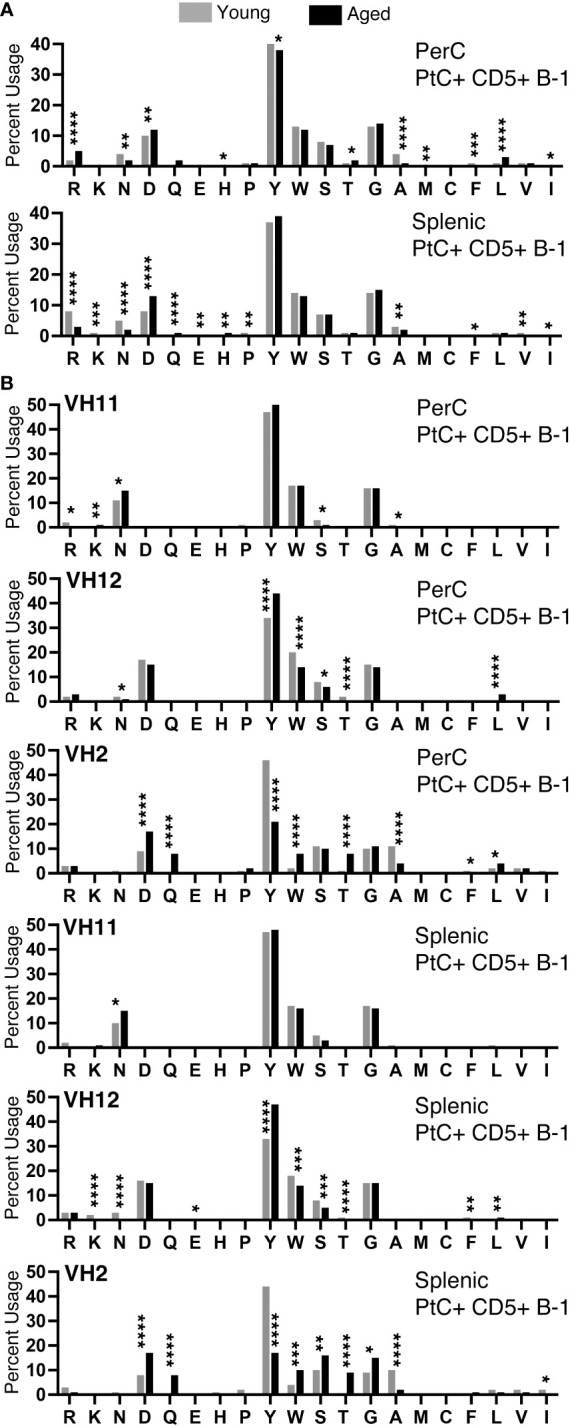
CDR-H3 amino acid distribution within the CDR-H3 changes with age. The percent of each amino acid used within the CDR-H3 was determined for each PtC+CD5+ B-1 cell subset as indicated. **(A)** Peritoneal and splenic PtC+CD5+ B-1 cells. **(B)** Peritoneal and splenic PtC+CD5+ B-1 cells utilizing VH11, VH12, or VH2. These results are based on sequences obtained from experiments performed in **Figure 2**. Results are based on 4 independent experiments with sequences combined from each independent experiment (n=11 for 3-month-old mice, n=15 for 23-26-month-old female mice). Statistics used: Chi-square test. Asterisks for p values: *p<0.05, **p<0.01, ***p<0.001, ****p<0.0001.

We examined the amino acid content of the CDR-H3 regions of PtC+CD5+ B-1 cells and found a predominance of tyrosine and glycine in cells from both the peritoneal cavity (PerC) and spleen (**Figure 7**). We observed many significant differences in amino acid content of PerC and splenic PtC+CD5+ B-1 cells between young and aged female mice (**Figure 7A**), the details of which are summarized in **Supplemental Figure 3**. When comparing these results to our previously published study of males, aged males displayed significantly fewer differences in amino acid content with age (24) as compared to aged females (**Figures 7A, B**). This distinction is even more apparent when examining sequences utilizing V_H_11, V_H_12, and V_H_2 (**Figure 7B** and (24)). Female sequences utilizing V_H_12 and V_H_2 had many significant changes in amino acid content with age (summarized in **Supplemental Figure 4**), which contrasts with the few changes observed in aged male mice (24). These results demonstrate differential selection of the PtC+CD5+ B-1 cell repertoire in males versus females over time.

We observed an overall preference for less hydrophobic and less charged amino acids in the CDR-H3 from both young and aged female PtC+CD5+ B-1 cells (**Figure 7A**). When examining sequences utilizing V_H_11, asparagine is preferentially used, whereas in sequences utilizing V_H_12 and V_H_2 aspartic acid usage is preferred (**Figure 7B**). These findings reflect previous studies showing DSP gene segments utilizing RF1 contain either asparagine or aspartic acid (49). Both splenic and PerC aged PtC+CD5+ B-1 cell sequences utilizing V_H_12 display an increase in tyrosine, whereas sequences utilizing V_H_2 display a decrease in tyrosine with age. These changes in CDR-H3 amino acid content could have implications for CD5+ B-1 cell function in the aged.

### 3.5 Biological sex influences the amount of circulating cell-free nucleic acid during aging

**Figure 8:**
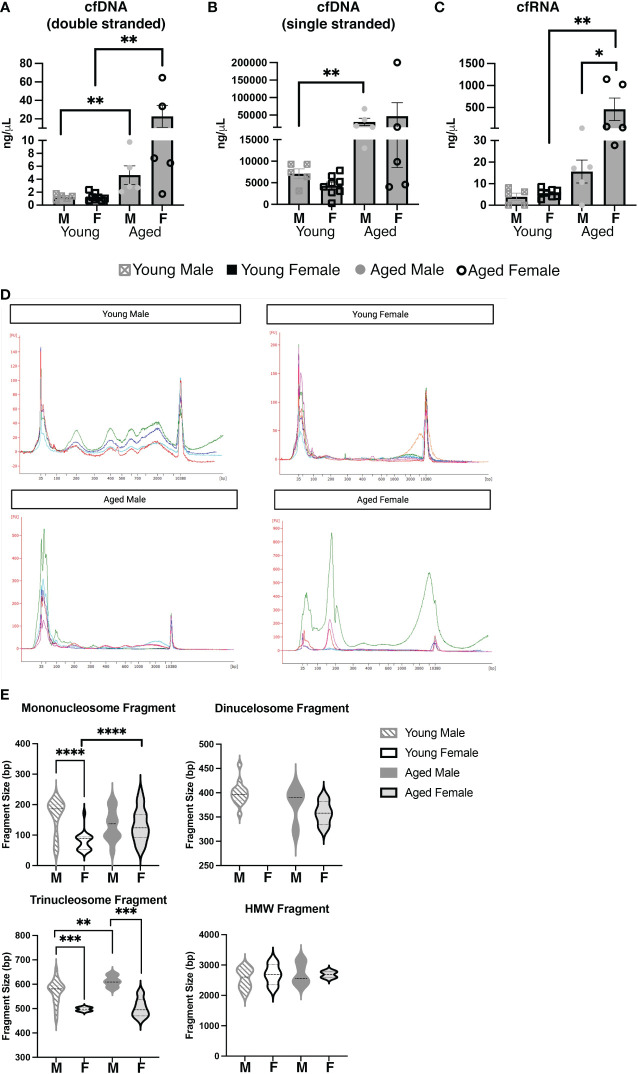
Aging and biological sex influences the levels and composition of serum cell-free nucleic acids. Serum was obtained from young (3-month-old) or aged (18-25-month-old) male and female BALB/c-ByJ mice (young male mice n=10, aged male mice n=10, young female mice n=7, aged female mice n=9). Cell-free (cf) nucleic acids were isolated from 200ul of each individual serum sample. **(A)** Double-stranded cfDNA, **(B)** single-stranded cfDNA, and **(C)** cfRNA were quantified using the Qubit 4. **(D)** cfDNA was measured on the Agilent 2100 Bioanalyzer using the HS DNA assay, and individual electropherograms for each sample were combined for each group (young and old males and females). **(E)** cfDNA fragment sizes for each of the three major cfDNA peaks (50–250, 250–450, 450–650) and the HMWcfDNA (~2300bp) from each trace were mapped with violin plots. Statistics used: Mann-Whitney test and Welch’s t-test (violin plots). Asterisks for p values: *p<0.05, **p<0.01, ***p<0.001, ****p<0.0001.

Considering our results demonstrating large differences in the serum and cellular repertoire of PtC-specific CD5+ B-1 cells in aged females as compared to young females and aged males, we hypothesized differences in antigenic load may be a driving factor. Recently, it was shown that Toll-like receptors (TLR) play a role in shaping the CD5+ B-1 cell repertoire, and TLRs sensing nucleic acids were specifically tied to anti-PtC responses (50). Cell-free nucleic acids (cfNCs) found in circulation are mainly the result of apoptosis (51). The most well characterized cell-free nucleic acid is DNA (cfDNA) (52), but many other types of cfNCs have been described including RNAs and mitochondrial DNA (53). After isolation of cfNC from the serum, we found a significant increase in double stranded cfDNA with age (**Figure 8A**). Levels of serum cfDNA increased from an average of 1.272 ng/μL in young males to 4.640 ng/μL in aged males. The increase in cfDNA in aged female serum was greater with young females having an average of 1.252 ng/μL and aged females 22.74 ng/μL of cfDNA (**Figure 8A**). Levels of single stranded cfDNA was significantly increased in aged male mice as compared to young male mice, and a non-statistically significant trend toward increase was observed in aged female mice (**Figure 8B**). When examining cfRNA, aged female mice displayed significantly increased levels of serum cfRNA as compared to both young females and aged males (**Figure 8C**).

We further explored the cell-free nucleic acid composition of these serum samples using a high sensitivity DNA assay for the Bioanalzyer (**Figures 8D, E**). Interestingly, young females had relatively low amounts of smaller (50-650bp) cfDNA but larger HMW cfDNA peaks as compared to young males (**Figure 8D**). The small cfDNA peaks were strong in aged females, as seen in both young and old males; however, in agreement with the quantitation from the Qubit (**Figures 8A-C**), the total amount of cfDNA is drastically increased in aged females as compared to the other groups (**Supplemental Figure 4**). Importantly, smaller (50-650bp) fragments have been shown to stem from apoptosis while larger fragments are thought to originate from necrosis (38, 54–56). To better explore the possible source of cfDNA from our samples, the fragment size was analyzed for the following three groups: 50-250 bp (corresponding to mononucleosome fragments), 250-450 bp fragments (corresponding to dinucleosome fragments), 450-650 bp fragments (corresponding to trinucleosome fragments) and high molecular weight (HMW) cfDNA fragments which were measured at 2,000-3,000 bp (**Figure 8E**). Total distribution and the average size of the fragment shows that the cfDNA products are different with both sex and age. In the 50-250bp fragment size, the most significant change is seen between young males and females: the average bp size of young males mononucleosome fragment is 161.5±12.27 bp while the young female is 79.26±6.895 bp (*p<0.0001*). This difference was not observed in aged males and females (132.5 bp vs. 133.3 bp, respectively, *p=0.9542*). These data suggest the origin of circulating cfDNA is primarily apoptosis of cells, but there are differences between females (of both age groups) as compared to males (**Figures 8D, E**). In total, the quantity and composition of cfDNA is significantly different between young and aged males and females. Together these results demonstrate the amount of cell-free nucleic acids in the serum of mice changes with age and/or biological sex.

## 4 Discussion

Our previous studies have demonstrated that protection against pneumococcal infection afforded by natural IgM is influenced by biological sex and age. In contrast to aged males, B-1 cell derived natural IgM obtained from aged females retains the ability to protect against *S. pneumoniae* infection (25). To explore how females are able to retain protective natural antibodies (NAbs) into old age we examined antigen specific NAbs in young and aged female mice. Specifically, we sought to determine whether sex and age influence CD5+ B-1 cells producing natural antibody specific for the phospholipid phosphatidylcholine (PtC), which is protective against sepsis (31).

We find an increase in the number of splenic PtC+CD5+ B-1 cells in aged females as compared to aged males, in correlation with 18 times more serum anti-PtC (DMPC) specific IgM in aged female vs. aged male mice. BCR repertoire analysis reflects this change as well. We found splenic PtC+CD5+ B-1 cell IgM maintains germline-like status in aged females more than aged males, suggesting that retention of protection in aged females is in part due to sex-related amino acid structural differences in the B cell receptor and corresponding antibody. Furthermore, we observed differences in the amino acid use within the antigen binding region of the antibody (CDR-H3 region) based on both sex and age. Changes in amino acid use within the CDR-H3 are indicative of selection over time. Increase in arginine use is associated with an increase in autoreactive antibodies (44, 57, 58). Herein, we demonstrate female peritoneal PtC+CD5+ B-1 cells have an increase in arginine use with age, suggesting this population could contribute to a corresponding increase in autoantibodies during aging. Together, our results demonstrate significant differences in the actual (serum PtC specific antibody) and available (cellular PtC specific BCRs) PtC repertoire in aged males and females.

It has been previously shown that changes in B cell repertoire such as D_H_ usage, J_H_ usage, N-region additions (germline status), and/or amino acid content within the CDR-H3 greatly influences the hydrophobicity of the CDR-H3 (45). Hydrophobicity is a characteristic of the CDR-H3, which plays a role in the interaction of the antibody and antigen. Hydrophobic CDR-H3 loops have been shown to be important for certain broadly neutralizing antibodies against HIV (43). The CDR-H3 loops of peritoneal cavity B-1 cell antibodies are characteristically hydrophobic (44). We found that as males and females age, the CDR-H3 regions change in hydrophobicity based on their location: splenic PtC+CD5+ B-1 cells increase remain hydrophobic while those in the peritoneal cavity increase in charge. While the trends in hydrophobicity with age are similar for aged males and females, we find female PtC+CD5+ B-1 cell IgM is more charged than male PtC+CD5+ B-1 cell IgM in both the spleen and peritoneal cavity. Importantly, the CDR-H3 of autoreactive antibodies is more charged than the CDR-H3 of non-autoreactive antibodies (44, 45). The results presented here are intriguing and consistent considering the higher prevalence of autoimmunity seen in females (59).

When comparing the repertoire of healthy aged mice to those with disease, there are differences in the utilization of specific CDR-H3 sequences (summarized in **Supplemental Figure 6**). Herein, we find peritoneal B-1 cells from aged females utilize AGDSYGYWYFDV (V_H_12/D_H_1-1/J_H_1) most frequently. In female B6*.Sle1.Sle2.Sle3* lupus-prone mice, peritoneal B-1 cells utilize AGDYDGYWYFDV (V_H_12/D_H_2/J_H_1) and MRYGNYWYFDV (V_H_11/D_H_2/J_H_1) most frequently (60), which are distinct from the CDR-H3 utilization we observe in the healthy aged female mice herein. In CD5+ B-1 cells obtained from aged male ApoE knockout mice, the most frequently utilized CDR-H3 is AGDYDGYWYFDV (V_H_12/D_H_2/J_H_1) (61), which is the same frequently utilized CDR-H3 seen in the lupus-prone mice. Interestingly, the frequently utilized CDR-H3 sequences observed in the aged utilize V_H_12, which has been previously shown to expand with age in an antigen-dependent manner (47). V_H_12 is known to recognize the phospholipid PtC (48). It is possible PtC could play a role in selection of the B cell repertoire over time. Interestingly, phospholipids have been shown to have direct effects on B cell function (62–64). Alterations in the regulation of PtC have been shown to be influenced by both age (65, 66) and sex (67, 68). Our data illustrating sex and age-related differences in V_H_12 utilization warrant further investigation regarding differences within the aging environment that influence the selection of B-1 cells and the natural antibodies they produce over time.

Overall, our BCR repertoire results suggest the antigenic environment of aged females may differ from that of aged male, and our analysis of cell-free DNA (cfDNA) supports that conclusion. The maintenance of CD5+ B-1 cells recognizing the phospholipid PtC is dependent on nucleic acid-sensing Toll-like receptors (TLR) (50). Aging is associated with increased cell senescence (69), decreased clearance of apoptotic cells (70), and higher levels of circulating cell-free nucleic acids (such as DNA, RNA, mtDNA, self and non-self-nucleic acids) (71, 72), which could be ligands for such nucleic acid sensing TLRs in the aged. Here, we report that cell-free nucleic acid levels differ in males and females during aging. In healthy individuals, cell-free nucleic acids are mainly derived from hematopoietic cells (39, 51), whereas, in disease states, an accumulation of cell-free nucleic acids results from the diseased tissue (40). cfDNA circulating in plasma has been an intensively investigated biomarker for the diagnosis of cancer, with specific cfDNA fragment sizes and sequences associated with specific forms of cancer (73–76). Here, we found that the size of the cfDNA fragment is associated with sex and age. We demonstrated a drastically different mononucleosome fragment size of cfDNA obtained from the serum of young male mice (162 bp) as compared to young female mice (79 bp), which changes with age to the same average length in both aged males and females (133 bp); however, aged females have much more cfDNA as compared to the other age and sex groups. Importantly, accumulation of cell-free nucleic acids might be one type of antigenic load that could play a role in the selection of PtC+ B-1 cells over time through nucleic acid sensing TLRs.

Our study highlights the need to further our understanding of how both the male and female aging environments impact the function and repertoire of B cells over time. As shown here, both age and biological sex influence PtC-specific antibodies, which provide protection against sepsis (31). Importantly, sex differences have been shown in patients with sepsis over the age of 50, where women have lower mortality than men (32). As such, it will be essential to fully grasp the influence of sex in the context of age to design effective vaccines and treat diseases prevalent in the aging population.

## Data availability statement

The datasets presented in this study can be found in online repositories. The names of the repository/repositories and accession number(s) can be found below: https://www.ncbi.nlm.nih.gov/genbank/, 2620705, 2620704, 2620702, and 2020696.

## Ethics statement

The animal study was reviewed and approved by Western Michigan University Homer Stryker M.D. School of Medicine institutional IACUC committee.

## Author contributions

SW performed experiments, analyzed data, interpreted data, and was a major contributor to writing the manuscript. NT performed experiments. MC aided in data interpretation, provided technical expertise, and edited the manuscript. NH performed experiments, analyzed data, interpreted data, and wrote and edited the manuscript. All authors contributed to the article and approved the submitted version.
