# Supplementary material for: Age-related changes in antigen-specific natural antibodies are influenced by sex
Source: Front Immunol. 2023 Jan 12;13:1047297. doi: 10.3389/fimmu.2022.1047297 (PMC9878317; doi:10.3389/fimmu.2022.1047297)
Supplement: Supplementary file 1 [file DataSheet_1.docx]

Supplementary Material


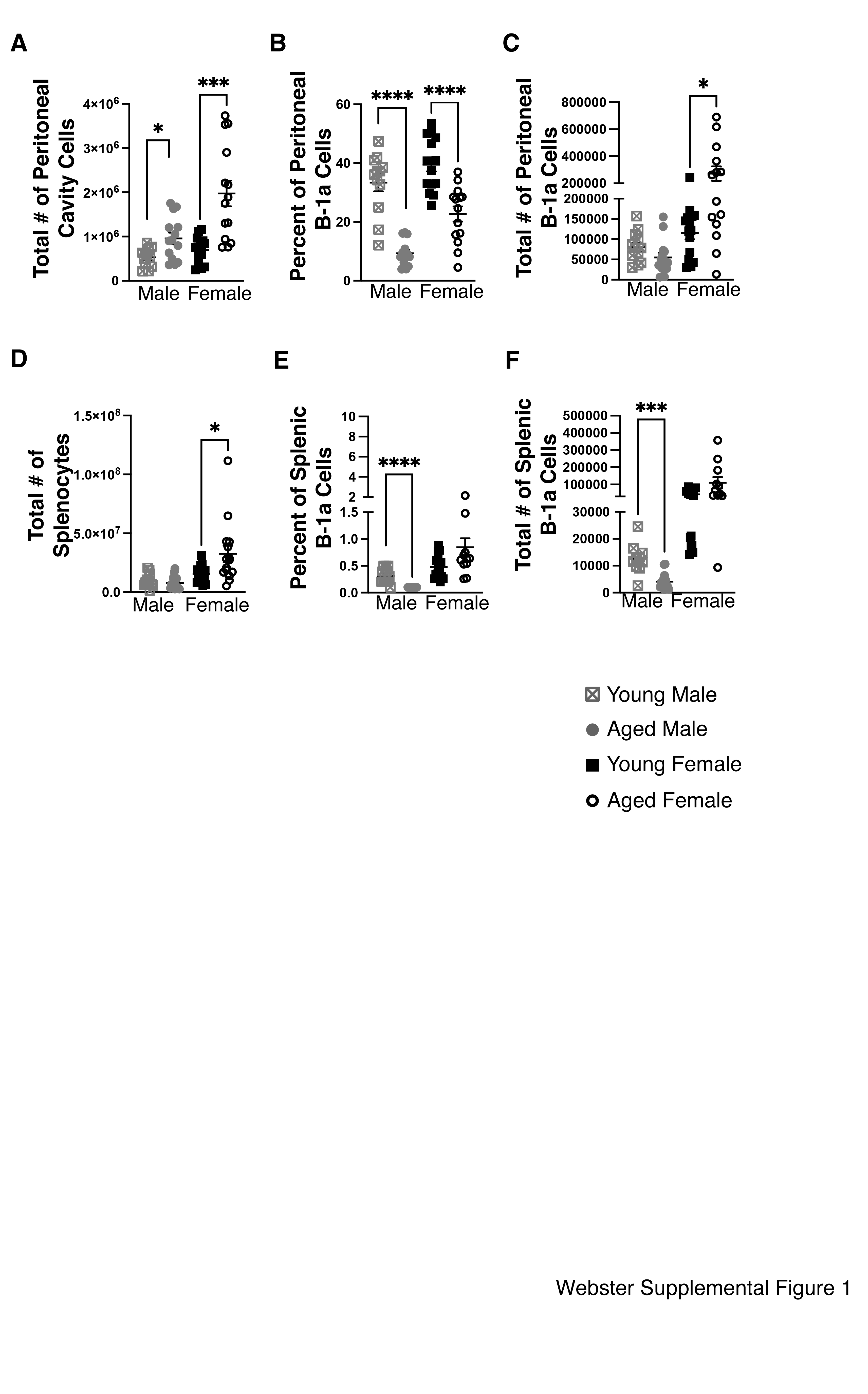


**Supplementary Figure 1. Number of CD5+ B-1 cells differs in male versus female mice.** CD5+ B-1 cells examined in the young (3-month-old) and aged (18-26-month-old), male and female, mice were assesed for percent and number. **(A)** The total number of peritoneal cavity cells, **(B)** the percent of live peritoneal lymphocytes staining postitive for CD5+ B-1 cells (B220^lo^CD5^+^CD19^hi^CD23^-^), **(C)** total number of peritoneal CD5+ B-1 cells, **(D)** the total number of splenocytes, **(E)** the percent of live splenocytes staining postitive for CD5+ B-1 cells (B220^lo^CD5^+^CD19^hi^CD23^-^), **(F)** total number of splenic CD5+ B-1 cells, Grey squares represent young male mice, grey circles represent aged male mice, black squares represent young female mice, and open black circles represent aged female mice. Results are based on 3 independent experiments. Values are displayed as the mean (±SEM) of individual mouse serum samples. Statistics used: Mann-Whitney test. Asterisks for p values: *p<0.05, **p<0.01, ***p<0.001, ****p<0.0001.

**
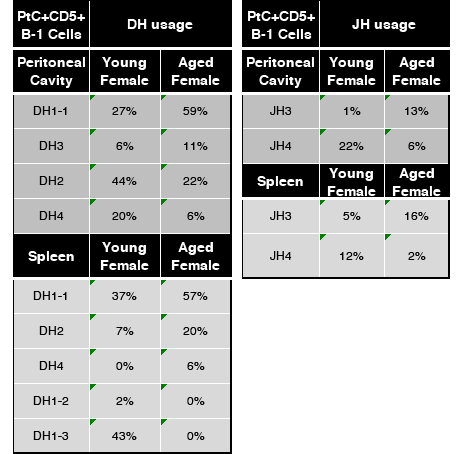
**

**Supplementary Figure 2. Summary of significant differences in D_H_ and J_H_ gene usage.** PtC+CD5+ B-1 cells were single-cell sorted from the peritoneal cavity or spleen of young and aged female BALB/c-ByJ mice (as presented in Figure 2). Summary of significant differences presented in Figure 4 of D_H_ and J_H_ gene segment usage. Results are based on 4 independent experiments with sequences combined from each independent experiment (n=11 for 3-month-old mice, n=15 for 23-26-month-old female mice). Statistics used: 2x2 and 2x4 chi-square test. Asterisks for p values: *p<0.05, **p<0.01, ***p<0.001, ****p<0.0001.


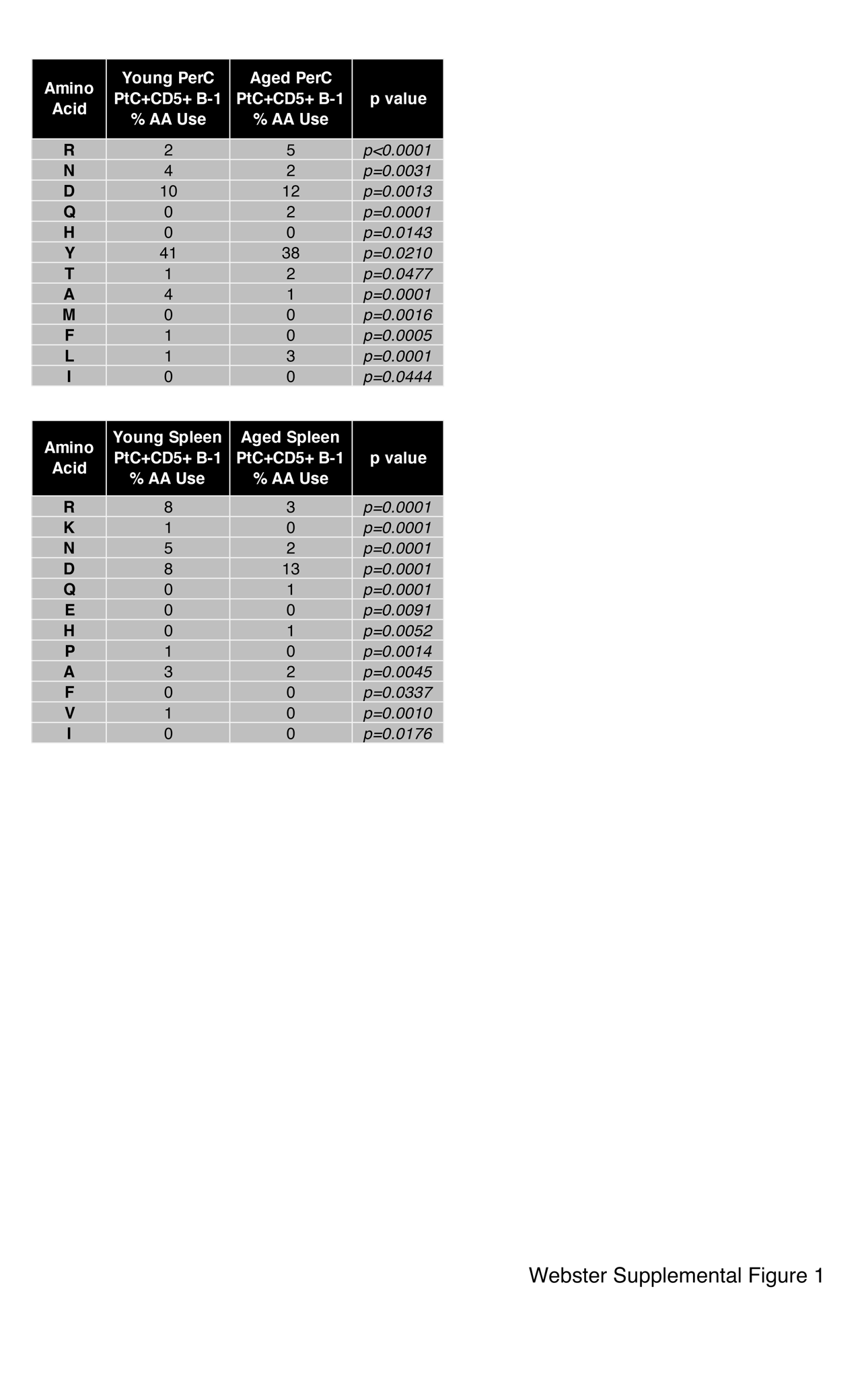


**Supplementary Figure 3.** The percent of each amino acid used within the CDR-H3 was determined for each PtC+ CD5+ B-1 cell subset as indicated. Statistics used: Chi-square.

**
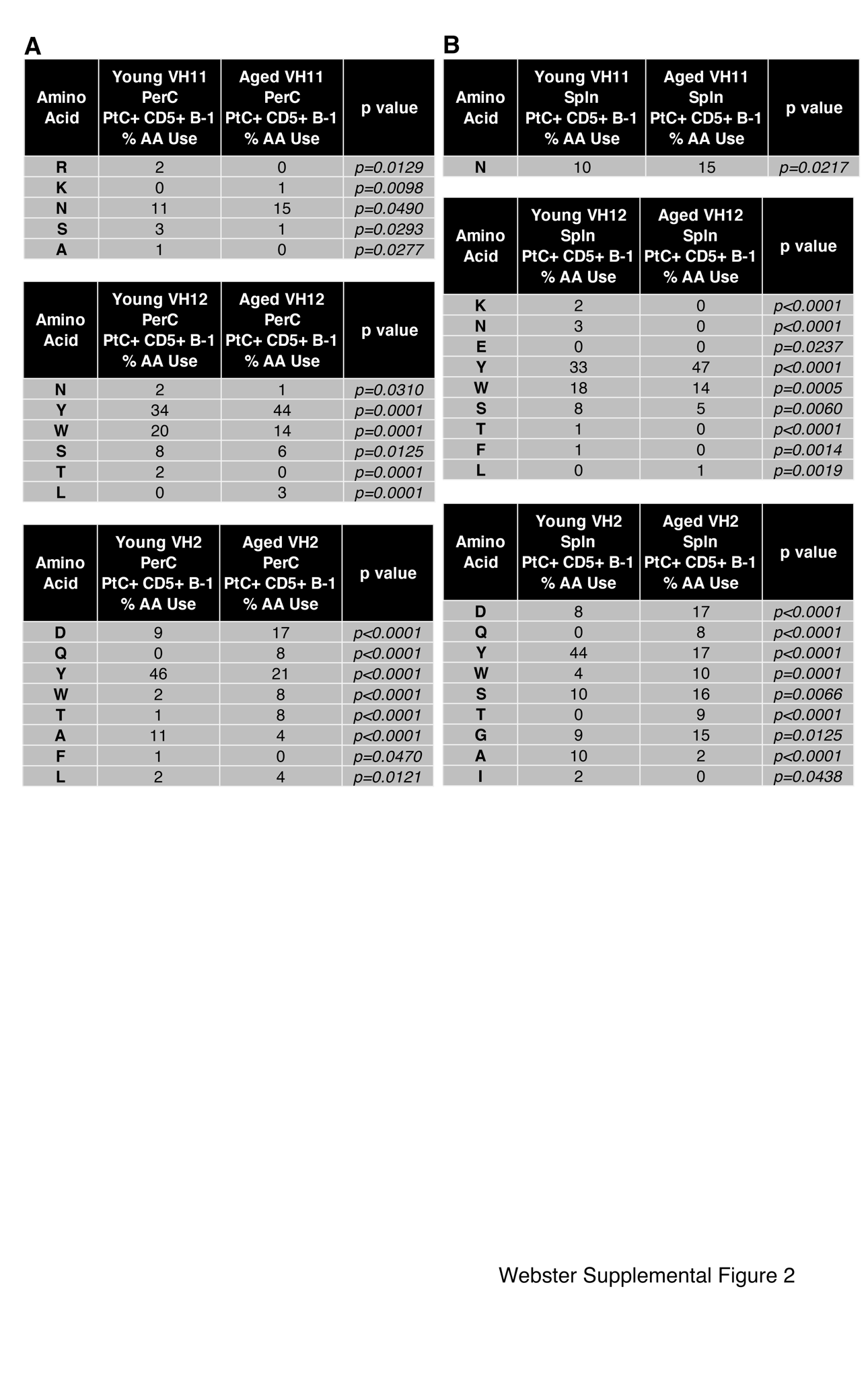
**

**Supplementary Figure 4.** The percent of each amino acid used within the CDR-H3 was determined for each PtC+ CD5+ B-1 cell subset utilizing a particular VH gene as indicated. Statistics used: Chi-square.

**Supplementary Figure 5.** The concentration of cell free DNA obtained from the serum of indicated mice analyzed using the Bioanalyzer.

**Supplementary Figure 6.** Most frequently utilized CDR-H3 sequences in aged mice of different health status. The source for color scheme of amino acids: RasMol Color scheme.
